# HBV pgRNA profiles in Chinese HIV/HBV coinfected patients under pre‐ and posttreatment: a multicentre observational cohort study

**DOI:** 10.1111/jvh.13704

**Published:** 2022-06-07

**Authors:** Ling Xu, Xiaodi Li, Lianfeng Lu, Xiaosheng Liu, Xiaojing Song, Yanling Li, Yang Han, Ting Zhu, Wei Cao, Taisheng Li

**Affiliations:** ^1^ Department of Infectious Diseases, Peking Union Medical College Hospital Chinese Academy of Medical Sciences & Peking Union Medical College Beijing China; ^2^ Center for AIDS Research Chinese Academy of Medical Sciences & Peking Union Medical College Beijing China; ^3^ Department of Infectious Diseases and Clinical Microbiology Beijing Chao‐yang Hosipital, Capital Medical University Beijing China; ^4^ Tsinghua University Medical College Beijing China

**Keywords:** cART, HBeAg loss, HBV DNA, HBV pgRNA, HIV/HBV coinfected patients

## Abstract

Data on hepatitis B virus (HBV) pregenomic (pgRNA) levels in HIV/HBV coinfected patients pre‐ and post‐combined antiretroviral therapy (cART) are limited. This study aimed to evaluate the distribution of HBV pgRNA levels in treatment‐naive coinfected patients and explore the changes that occur after the initiation of cART by examining patients from multicentre cohort studies performed in China. We included HIV/HBV coinfected subjects from the China AIDS Clinical Trial cohorts established from 2008 to 2014. Clinical and serological markers of HIV and HBV infection and biochemical data were acquired at baseline and after 96 and 240–480 weeks of cART. The correlations between HBV pgRNA and HBV DNA levels as well as HBsAg levels were calculated using Spearman's bivariate correlation analysis, and multivariate regression analysis was performed to determine factors associated with undetectable HBV pgRNA levels before cART and HBeAg loss after cART. A total of 132 HIV/HBV coinfected patients were enrolled, and 100 individuals were HBeAg‐negative. A total of 34.4% (32/93) of patients were positive for HBV pgRNA, and the median HBV pgRNA level was 4.92 (IQR: 4.21–6.12) log_10_ copies/mL before cART. The median HBV pgRNA level was significantly lower in HBeAg‐negative individuals than in HBeAg‐positive individuals (4.22 (IQR: 2.70–4.84) log_10_ copies/mL vs. 5.77 (IQR: 4.63–6.55) log_10_ copies/mL, *p* = 0.002). HBV pgRNA was moderately correlated with HBsAg (*r* = 0.594, *p* = 0.001), and positively associated with HBV DNA (*r* = 0.445, *p* = 0.011). The factors independently associated with undetectable HBV pgRNA level before cART were HBV DNA (OR: 5.61, 95% CI: 1.50–20.96, *p* = 0.01) and HBeAg status (OR: 5.95, 95% CI: 1.52–23.25, *p* = 0.01). A total of 87.5% (28/32) of patients were followed for a median duration of 138 (IQR: 54–240) weeks, and the HBV pgRNA levels became undetectable in seven patients. The 132 patients were observed for 695.5 person‐years, and no HBsAg loss occurred. Thirteen individuals achieved HBeAg loss, four patients had undetectable levels of HBV pgRNA pre‐cART, and the level of six individuals became undetectable during the 48‐week (IQR: 48–264) follow‐up period. HBeAg status was significantly associated with HBV pgRNA level in HIV/HBV coinfected patients pre‐ and post‐cART. Additionally, undetectable HBV pgRNA level may be associated with HBeAg loss after cART.

**Abbreviations**
ALT: alanine transaminase
AST: aspartate aminotransferase
CACT: China AIDS Clinical Trial
CHB: chronic hepatitis B
cART: combined antiretroviral therapy
cccDNA: covalently closed circular DNA
CTLs: cytotoxic T lymphocytes
HCC: hepatic cell carcinoma
HBV: hepatitis B virus
IQR: interquartile range
LLD: low limit of detection
TDF: tenofovir disoproxil fumarate
Tbil: total bilirubin

## 1 INTRODUCTION

HIV/HBV coinfection is fairly common in clinical practice due to shared transmission routes. The prevalence of coinfection is 8.4% worldwide^1^ and 9.5% in China.^2^ It has been reported that the coinfected prevalence is higher in populations where vaccination is not available such as sub‐Saharan.^1^ HIV/HBV coinfected patients are characterized by accelerated progression of liver disease and increased liver‐associated mortality.^3^ Although anti‐HBV regimen‐containing combined antiretroviral therapy (cART) has effectively suppressed HIV and HBV replication, morbidity and mortality remain significantly higher in coinfected patients than in those infected with HIV alone.^4^

Previous studies indicated that the HBV DNA suppression rate after 48 weeks of cART was 78.8% in coinfected patients,^5^ and 98.5% had undetectable levels of HBV DNA by year 5 of TDF‐inclusive cART.^6^ Unfortunately, cART is not able to eliminate covalently closed circular DNA (cccDNA), which is the main transcriptional template of HBV. Consequently, the level of HBV DNA rebounds once the treatment is discontinued in many chronic hepatitis B (CHB) patients. Therefore, monitoring the cccDNA levels plays an important role in evaluating the therapeutic efficacy and estimating the treatment endpoint. Intrahepatic cccDNA monitoring is conducted by liver biopsy, which is not widely used in clinical practice due to certain disadvantages, such as its invasiveness, an inadequate specimen size, interobserver variability and potential complications.^7^

Hepatitis B virus pgRNA is thought to be a transcription product of HBV and used as a plasma marker for infection outcome and for hepatic cell carcinoma (HCC).^8^, ^9^, ^10^, ^11^, ^12^ Patients who remain HBV pgRNA‐positive after treatment have an increased risk of viral rebound when the treatment is discontinued.^12^ Detectable HBV pgRNA is associated with HCC development in CHB patients receivingantiviral therapy.^11^ Unfortunately, the HBV pgRNA distribution in HIV/HBV coinfected patients has not been well elucidated. These HIV‐associated immunodeficiency patients are known to exhibit depletion of HBV‐specific cytotoxic T lymphocytes (CTLs) pre‐ and post‐cART, which may impact the control of HBV replication.^13^, ^14^ This implies that coinfected patients may display different characteristics of viral markers from HBV monoinfection patients and further study is necessary to explore the levels of potential serological markers, such as HBV pgRNA, in coinfected patients. To date, we found that only a cross‐sectional study demonstrated that there was no significant difference in the levels of HBV pgRNA between treatment‐naive individuals with HBV monoinfection and those with HIV/HBV coinfection. HBV pgRNA levels were strongly correlated with HBV DNA levels in coinfected patients.^15^ However, the small sample size and some limitations inherent from the single‐centre nature of that study make it difficult to fully understand the HBV pgRNA levels in treatment‐naive HIV/HBV coinfected patients. Moreover, no study has been conducted to demonstrate the changes in HBV pgRNA levels after cART in coinfected patients.

Additionally, highly potent tenofovir disoproxil fumarate (TDF)‐containing cART is able to effectively suppress HBV replication in either HBV‐infected or HIV/HBV coinfected patients. However, long‐acting drugs such as cabotegravir and rilpivirine, which do not contain any active anti‐HBV agents, are noninferior to standard TDF combined with 3TC‐containing therapy for maintaining the HIV RNA suppression rate.^16^, ^17^ It is worth considering whether HIV/HBV coinfected patients who have been treated with anti‐HBV regimens‐including cART for a long time could discontinue 3TC and TDF and commence on cabotegravir and rilpivirine therapies. Based on this fact, it is essential to understand the effect of long‐term TDF with and without 3TC‐containing treatment in HIV/HBV coinfected patients, which may provide important clues for the further clinical management of HIV/HBV coinfected patients. Hence, the primary aims of this multicentre cohort study were to evaluate the levels of HBV pgRNA pre‐ and post‐cART and explore their association with the long‐term prognoses in HIV/HBV coinfected patients.

## 2 METHODS

### 2.1 Study population

We reviewed cART‐naive HIV/HBV coinfected patients from the following prospective, multicentre cohort studies: the China AIDS Clinical Trial (CACT) 0810 and CACT1215 (ClinicalTrials. gov identifiers: NCT00872417 and NCT01844297). As previously described,^2^ HIV‐infected individuals in CACT0810 were enrolled between November 2008 and January 2010. Eligibility criteria included^1^ age between 18 and 65 years,^2^ CD4^+^ cell count lower than 350 cells/μL, and^3^ cART naivety. Participants in CACT1215 were enrolled between August 2012 and September 2014. CACT1215 had the same inclusion criteria as CACT0810, except that the CD4^+^ cell count threshold was 500 cells/μL. Subjects received zidovudine or stavudine in combination with lamivudine and nevirapine or efavirenz in the CACT0810 cohort and tenofovir combined with lamivudine and efavirenz or nevirapine in the CACT1215 cohort. The CACT1315 was an extension of the above two studies beyond the initial 96‐week follow‐up period to the time of the present analysis, so these data in this study were collected from 2008 to 2020.

Subjects visited local medical centres for clinical evaluation and blood collection before cART (baseline) and at the following weeks after cART initiation at 4, 8, and 12 weeks and then every 12 weeks. In this study, we retrieved demographic and clinical data including age, sex, HIV transmission route, alanine transaminase (ALT), aspartate aminotransferase (AST), total bilirubin (Tbil), CD4^+^ cell count, HIV RNA, HBsAg, HBeAg and anti‐HCV serostatus at baseline (within 4 weeks of cART initiation), 96 weeks, and 240–480 weeks. In HBsAg‐positive patients, HBV DNA and HBV pgRNA levels were measured. Participants were defined as having HIV/HBV coinfection if they were HBsAg‐positive and anti‐HCV‐negative. In this study, subjects who were both HBsAg and anti‐HCV‐positive were excluded (*n* = 6).

### 2.2 Measurement of HIV RNA and HBV DNA levels

Plasma samples were separated from whole blood by centrifugation within 4 h of collection and stored at −80°C until testing. HIV RNA and HBV DNA levels were quantified with a COBAS AmpliPrep/TaqMan48 real‐time PCR system (Roche Molecular Systems, Pleasanton, CA, USA) in the Department of Infectious Disease Laboratory at PUMCH according to the manufacturer's instructions. The linear ranges of HIV RNA and HBV DNA were 50–1 000 000 copies/mL (1.70–6.00 log_10_ copies/mL) and 20–170 000 000 IU/mL (1.30–8.23 log_10_ IU/mL), respectively. HBV DNA suppression was defined as <20 IU/mL.

### 2.3 Measurement of serum HBV pgRNA

The extraction of HBV pgRNA from 200 μl serum of HIV/HBV coinfected individuals and DNase I treatment were performed using commercial kits (Beijing Hotgen Biotech. Co. Ltd) following the manufacturer's instructions. The levels of HBV pgRNA were detected with the HBV pgRNA One‐Step RT‐qPCR Kit (Beijing Hotgen Biotech.Co. Ltd) as previously described.^12^, ^18^ During quantification, five gradients of quantification standards were used, with 10^3^, 10^4^, 10^5^, 10^6^ and 10^7^ copies/mL, respectively. The minimum and maximum detection limits of this method were 300 copies/mL and 10^8^ copies/mL, respectively.

### 2.4 Ethics statement

The Institutional Review Board of Peking Union Medical College Hospital (PUMCH) approved the parent studies and each participant provided written informed consent.

### 2.5 Statistical analysis

Analyses were performed using SPSS 23.0 (IBM Corp, Armonk, NY, United States). Descriptive statistics are presented as the mean with standard deviation (SD) or median (M) with interquartile range (IQR). Student's *t*‐test was used for comparisons of parametric data, and the Mann–Whitney *U* test was used for comparisons of noncategorical variables. Categorical variables were analysed by the chi‐squared test or Fisher's exact test. Associations between two variables were calculated using Spearman's bivariate correlation analysis. A logistic regression model and Cox regression model were applied to identify factors closely related to undetectable HBV pgRNA levels and HBeAg seroconversion, respectively. Statistical significance was defined as a *p* value less than 0.05.

## 3 RESULTS

### 3.1 Patients characteristics

**FIGURE 1:**
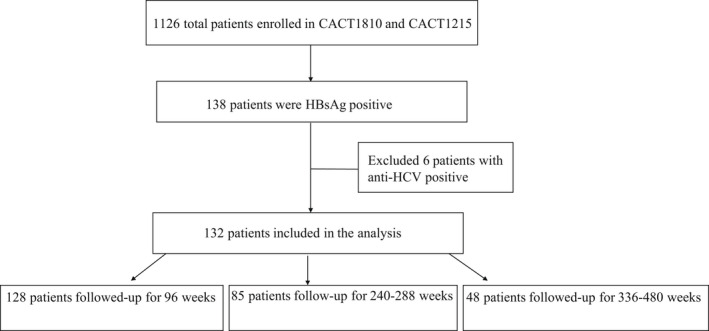
Flow of patients through the screening process

From the patients enrolled in the parent studies, we identified 138 participants who were positive for HBsAg in the database. An additional six patients were excluded because of anti‐HCV positivity and 132 HIV/HBV coinfected individuals were ultimately included (Figure 1).

The median age of the 132 included participants was 35 (IQR: 31–45) years old, and 78.8% were male. Most of the patients were infected via sexual transmission. The median CD4^+^ cell count was 213 (IQR: 127–309) cells/μL, and the median HIV RNA load was 4.69 (IQR: 4.21–5.15) log_10_ copies/mL. Forty‐five individuals received 3TC‐based regimens, and 75 received 3TC combined with TDF‐based regimens.

Notably, 100 patients were HBeAg‐negative, and 32 were HBeAg‐positive. The HBV DNA, HBsAg and ALT levels in HBeAg‐negative individuals were significantly lower than those in HBeAg‐positive patients. HIV RNA and CD4^+^ cell counts were comparable in the two groups (Table 1).

**TABLE 1:** Baseline characteristics of enrolled patients

	HBeAg(−) (*N* = 100)	HBeAg(+) (*N* = 32)	*p*
Sex, *n* (%)			
Men	77 (77.0%)	27 (84.38%)	<0.001
Age (years, IQR)	36 (31–45)	33.5 (31–44)	0.694
Transmission route, *n* (%)			
Homosexual	24 (24.0%)	8 (25.0%)	<0.001
Heterosexual	64 (64.0%)	18 (56.25%)	
Bisexual	3 (3.0%)	0 (0%)	
Blood	3 (3.0%)	0 (0%)	
Others/unknown	6 (6.0%)	6 (18.75%)	
CD4^+^ T cell count (cells/μL)	229 (146–312)	171 (88–285)	0.109
CD8^+^ T cell count (cells/μL)	784 (492–1064)	803.5 (568.3–1230.3)	0.340
CD4^+^/CD8^+^ ratio	0.27 (0.18–0.42)	0.19 (0.10–0.33)	0.017
HIV RNA (log10 copies/mL)	4.63 (4.2–5.14)	4.76 (4.4–5.33)	0.69
HBV DNA (log10 IU/mL)	3.02 (1.95–4.73)	8.04 (7.59–8.04)	<0.001
qHBsAg (log_10_ IU/mL)	3.17 (2.28–3.69)	4.79 (4.57–5.0)	<0.001
Syphilis coinfection, *n* (%)	12 (12.0%)	6 (18.75%)	<0.001
ALT (IU/L, IQR)	28 (20–39.3)	36.35 (30–47)	0.004
AST (IU/L, IQR)^a^	27.6 (23–37.5)	28.5 (24.0–36.2)	0.601
TBil (mg/dL, IQR)	10.9 (8.3–14.6)	11.3 (8.9–15.5)	0.502
APRI (IQR)	0.37 (0.29–0.55)	0.35 (0.28–0.49)	0.461
FIB4 (IQR)	1.02 (0.77–1.58)	0.87 (0.63–1.57)	0.498
cART regimens^b^			
3TC + AZT/d4T + EFV/NVP	32 (32.0%)	13 (40.63%)	<0.01
3TC + TDF + EFV/NVP/LPV/r	56 (56.0%)	19 (59.38%)	
Unknown	12 (12.0%)	0 (0%)	

^a^ 93 patients AST data available.

^b^ 120 patients cART regimens available.

### 3.2 HBV DNA response during the follow‐up period

**FIGURE 2:**
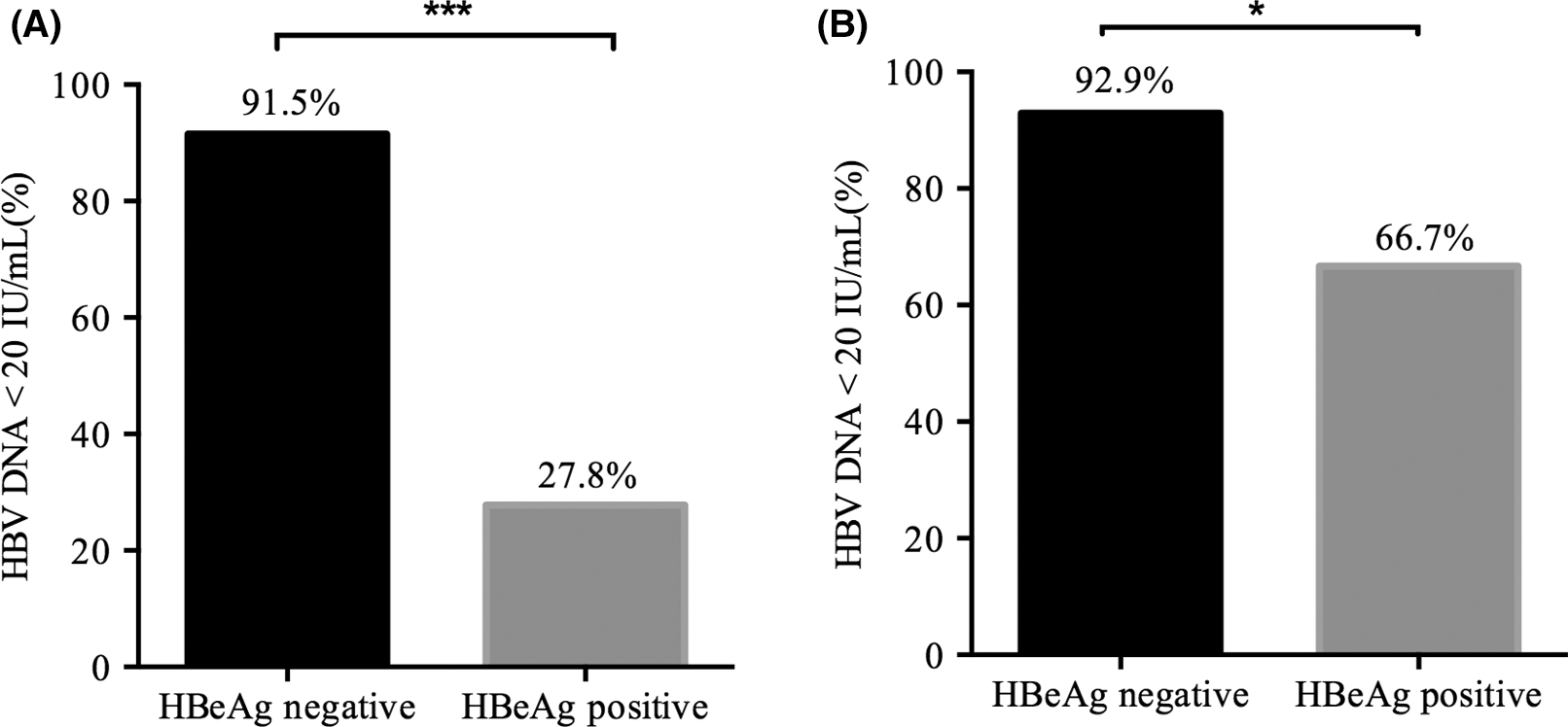
Rate of HBV DNA suppression during cART treatment. The rate of HBV DNA suppression after 96 (A) and 240–480 weeks (B) of cART in HIV/HBV coinfected patients stratified by HBeAg status

Hepatitis B virus DNA suppression was achieved in 76.62% (59/77) of individuals after 96 weeks of cART. Additionally, the HBV DNA suppression rates were higher in HBeAg‐negative participants than in HBeAg‐positive participants (91.5% (54/59) vs. 27.8%, (5/18), (*p* < 0.001)] (Figure 2A). A total of 88.4% (61/69) of individuals achieved effective HBV DNA suppression after a median duration of 240 (IQR: 240–312) weeks of cART. In HBeAg‐negative participants, the HBV DNA suppression rate was 92.9% (52/56), which was higher than that in HBeAg‐positive participants (66.7%, 8/12) (*p* = 0.028) (Figure 2B). Notably, HBV DNA suppression rates were higher in the TDF and 3TC‐based cART groups than in the 3TC monotherapy group (96.9% vs. 80.0%) (*p* = 0.056).

### 3.3 HBV pgRNA levels in cART‐naive HIV/HBV coinfected patients

**FIGURE 3:**
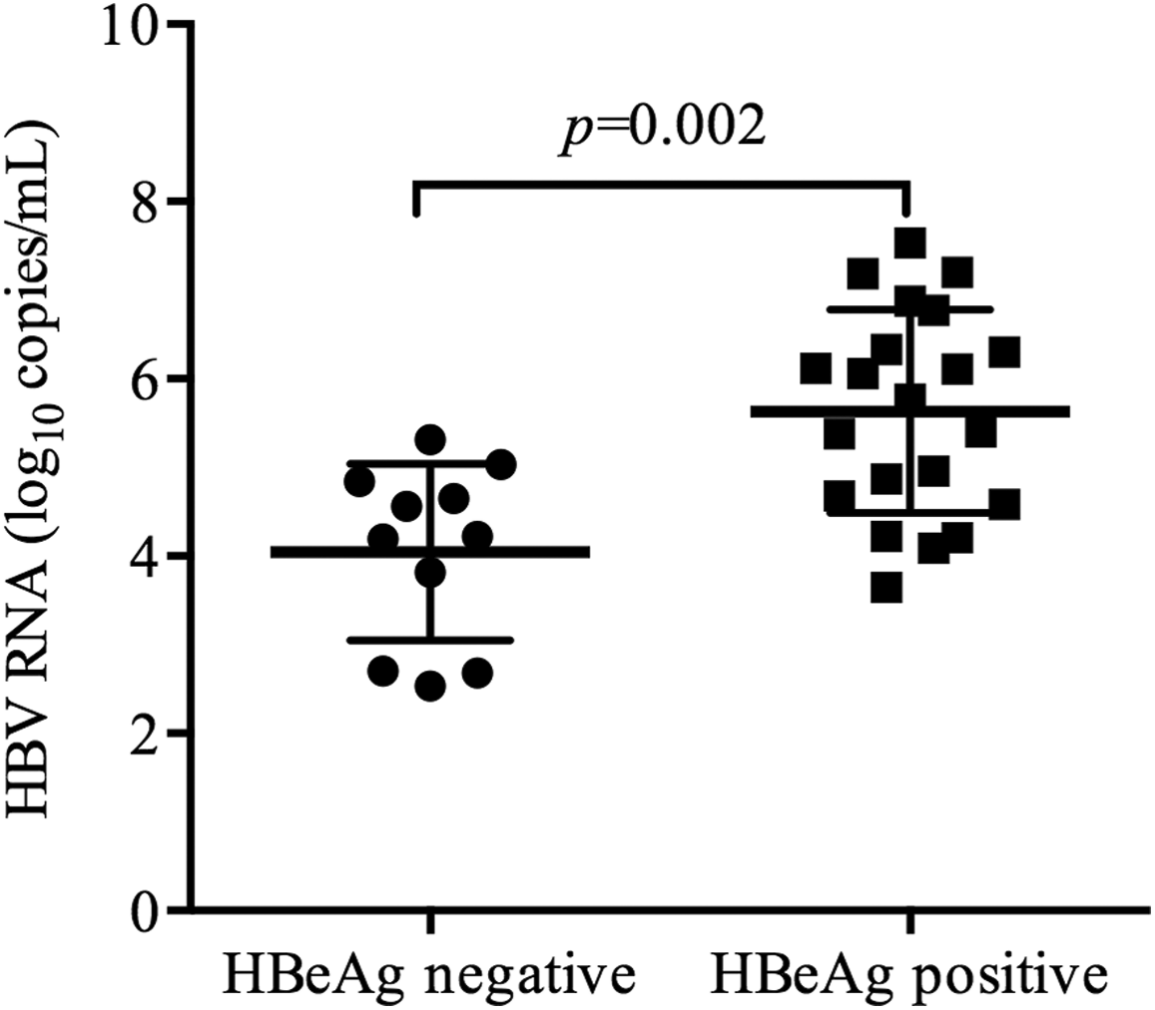
HBV pgRNA levels before cART. HBV pgRNA levels are shown in cART‐naive HIV/HBV coinfected patients

**FIGURE 4:**
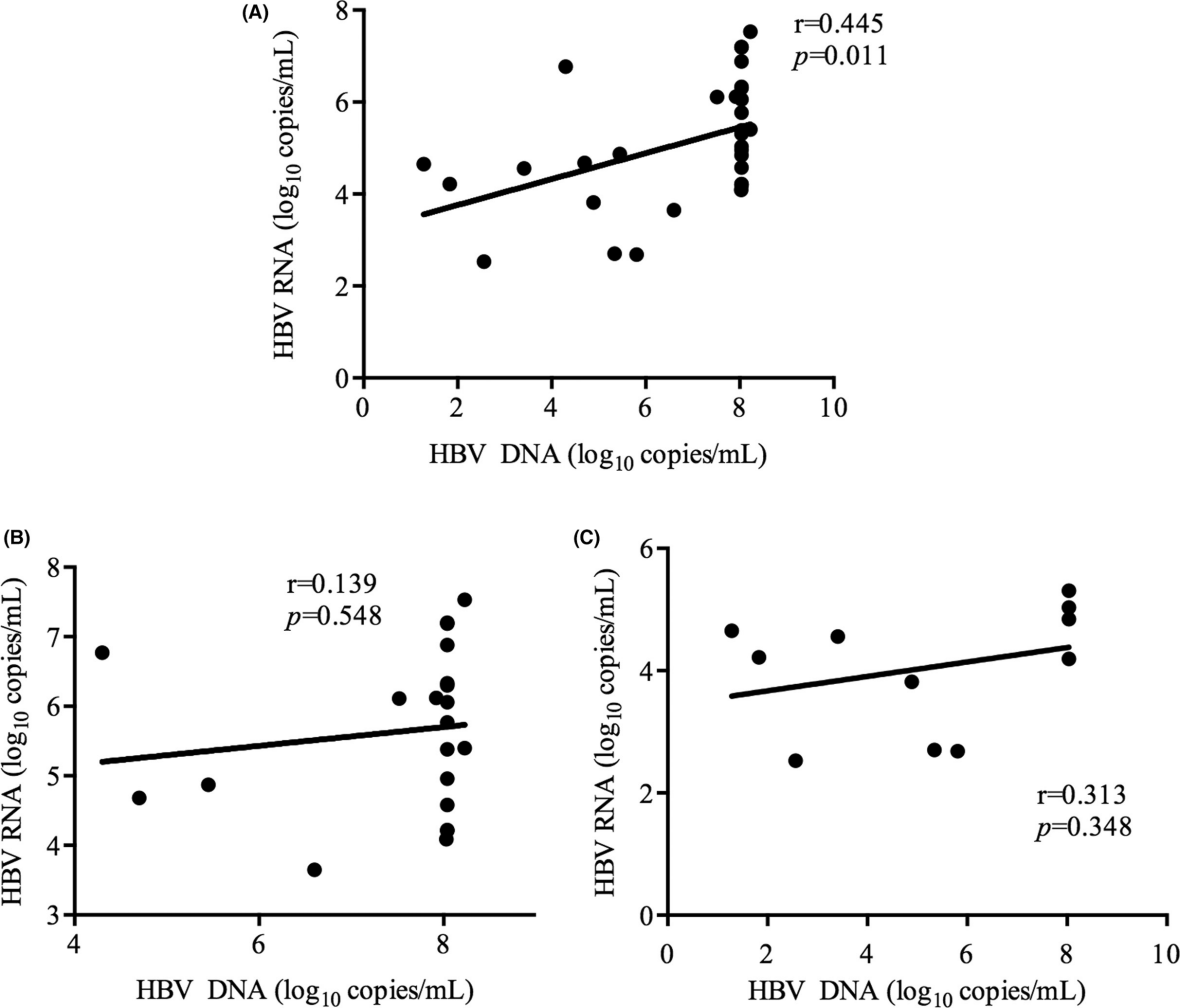
Correlations between HBV pgRNA and HBV DNA. The association between HBV pgRNA and HBV DNA in enrolled HIV/HBV coinfected patients (A), HBeAg‐positive coinfected patients (B) and HBeAg‐negative coinfected patients (C) before treatment

**FIGURE 5:**
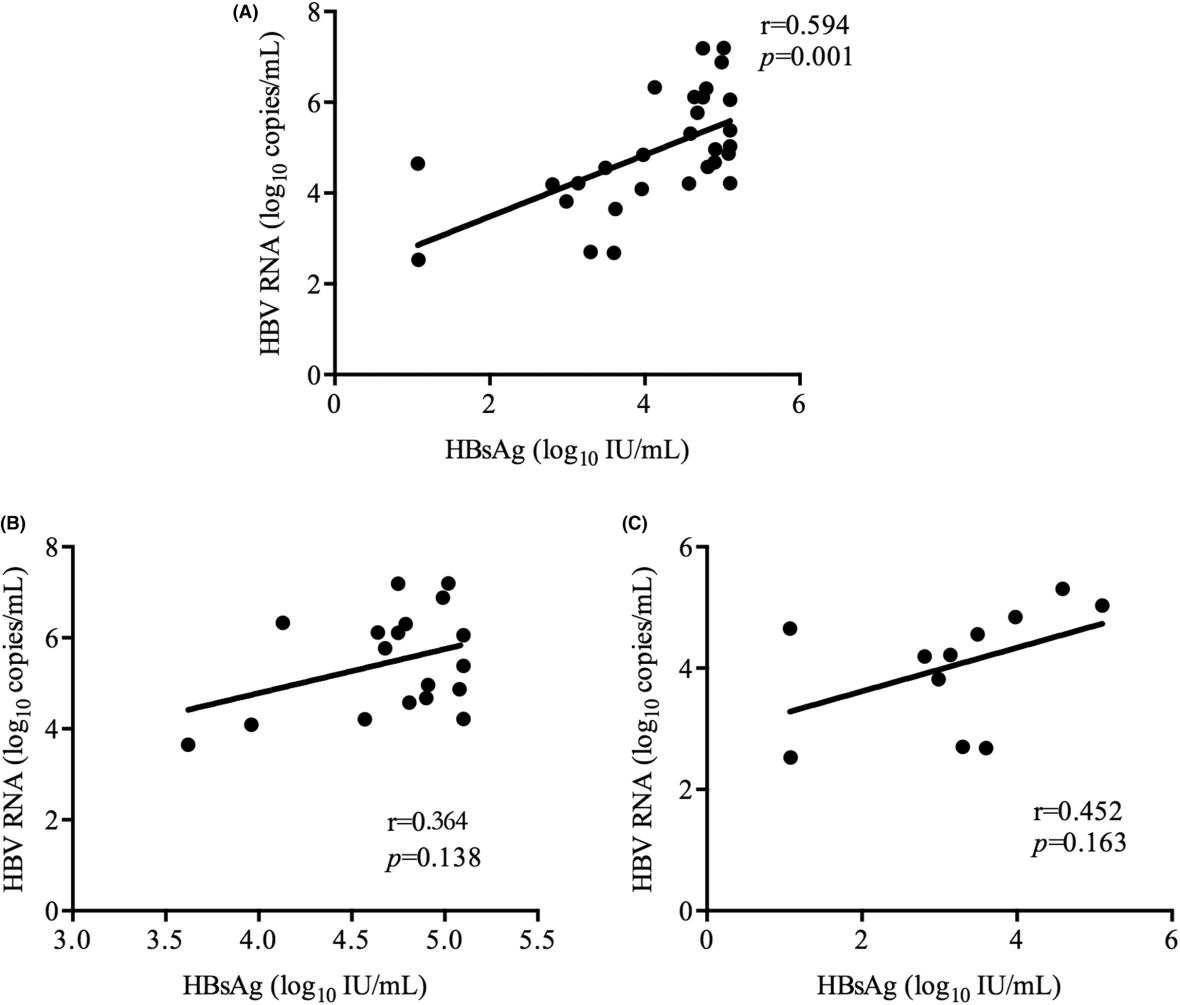
Correlations between HBV pgRNA and HBsAg. The association between HBV pgRNA and HBsAg in enrolled HIV/HBV coinfected patients (A), HBeAg‐positive coinfected patients (B) and HBeAg‐negative coinfected patients (C) before treatment

Hepatitis B virus pgRNA results were available for 93 treatment‐naive HIV/HBV coinfected patients, of whom 26 were HBeAg‐positive. HBV pgRNA was detectable in 32 patients, and the median level was 4.92 (IQR: 4.21–6.12) log_10_ copies/mL. Of the 32 patients, 21 were HBeAg‐positive and 11 were HBeAg‐negative. The HBV pgRNA levels were higher in HBeAg‐positive patients than that in HBeAg‐negative patients (5.77 (IQR: 4.63–6.55) log_10_ copies/mL vs. 4.22 (IQR: 2.70–4.84) log_10_ copies/mL) (*p* = 0.002) (Figure 3). The median HBV DNA level in these 32 patients was 8.04 (IQR: 5.37–8.04) log_10_ copies/mL. Further analysis showed that HBV pgRNA levels were positively correlated with the HBV DNA and HBsAg levels (*r* = 0.445, *p* = 0.011) (Figure 4A), (*r* = 0.594, *p* = 0.001) (Figure 5A). However, these correlations were not observed when patients were stratified by HBeAg status (Figure 4B, C, 5B, C). Furthermore, the HBV pgRNA levels were not associated with the HIV viral load (*r* = −0.001, *p* = 0.995), CD4^+^ cell counts (*r* = 0.02, *p* = 0.912) or CD4^+^/CD8^+^ ratio (*r* = −0.121, *p* = 0.509) at baseline.

The serum HBV pgRNA level was under the low limit of detection (LLD) in 61 individuals, of whom 56 were HBeAg‐negative, and 5 were HBeAg‐positive. The proportion of patients with undetectable HBV pgRNA levels was significantly lower in the HBeAg‐positive group than in the HBeAg‐negative group. As expected, compared to patients with HBV pgRNA levels below the LLD, HBV pgRNA‐positive individuals had higher HBV DNA and HBsAg levels. However, the HIV RNA levels, CD4^+^ cell count and CD4^+^/CD8^+^ ratio was comparable between the two groups (Table 2).

**TABLE 2:** Comparation of characteristics between HBV pgRNA‐positive and HBV pgRNA‐negative patients pre‐cART

		HBV pgRNA(+) (*N* = 32)	HBV pgRNA(−) (*N* = 61)	*p*
Sex, *n* (%)	Men	26 (81.25%)	49 (80.33%)	0.915
Age (years, IQR)		36 (31–45)	37 (30–47)	0.984
Pre‐cRT	HBV DNA (IU/mL)	8.04 (5.37–8.04)	3.02 (2.00–4.34)	<0.001
	HBsAg (log_10_ copies/mL)	4.64 (3.55–4.95)	3.07 (2.17–3.68)	<0.001
	HBeAg‐positive	21 (65.63%)	5 (8.20%)	<0.001
	HBeAg‐negative	11 (34.38%)	56 (91.80%)	
	CD4^+^ T cell count (cells/μL)	171 (69–308)	242 (158–313)	0.128
	CD4^+^/CD8^+^ ratio	0.22 (0.13–0.36)	0.25 (0.15–0.35)	0.339
	HIV RNA (log_10_ copies/mL)	4.61 (4.16–5.12)	4.73 (4.34–5.16)	0.494
cART	3TC‐based	10 (31.25%)	12 (19.67%)	0.228
	3TC + TDF‐based	22 (68.75%)	48 (78.69%)	

We further studied the factors associated with HBV pgRNA levels below the LLD using multivariate logistic regression analysis. The factors independently associated with undetectable HBV pgRNA levels were HBV DNA (OR: 5.61, 95% CI: 1.50–20.96, *p* = 0.01) and HBeAg status (OR: 5.95, 95% CI: 1.52–23.25, *p* = 0.01) at baseline (Table 3).

**TABLE 3:** Factors associated with undetectable HBV pgRNA in cART‐naive coinfected patients

	Univariable	*p*	Multivariable	*p*
	OR (95% CI)		OR (95% CI)	
Gender				
Male	Reference			
Female	0.85 (0.29–2.51)	0.771		
Age (year)	0.997 (0.95–1.04)	0.903		
HBV DNA (log_10_ IU/mL)	12.38 (4.22–36.32)	<0.001	5.61 (1.50–20.96)	0.01
HBsAg (log_10_ IU/mL)	3.14 (1.78–5.54)	<0.001		
HBeAg status				
Negative	Reference		Reference	
Positive	21.28 (6.64–68.90)	<0.001	5.95 (1.52–23.25)	0.01
cART				
3TC‐based	Reference			
3TC + TDF‐based	0.55 (0.21–1.46)	0.231		
CD4^+^ T cell count				
<200 cells/μL	Reference			
>200 cells/μL	0.54 (0.23–1.28)	0.163		
HIV RNA				
<5 log_10_ copies/mL	Reference			
>5 log_10_ copies/mL	0.91 (0.36–2.28)	0.835		

### 3.4 HBV pgRNA response during the follow‐up period

**FIGURE 6:**
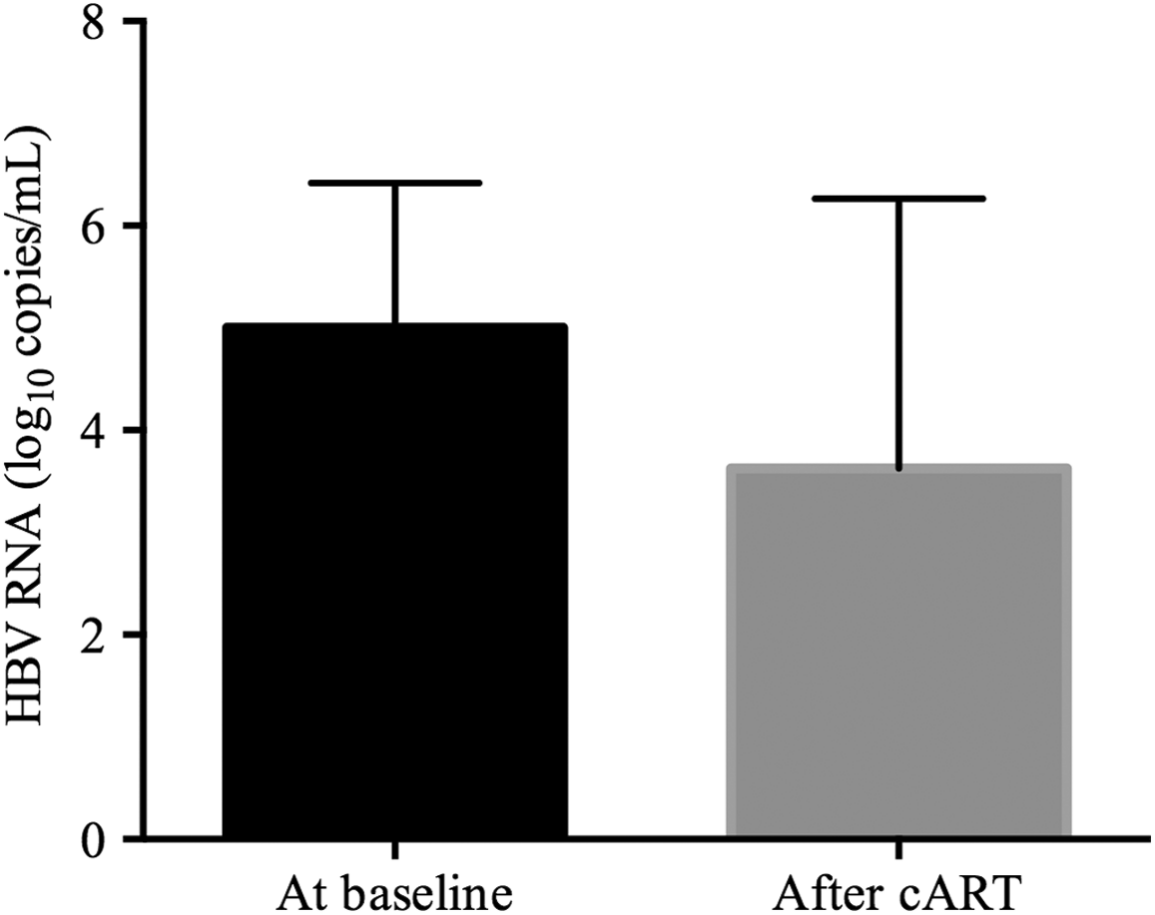
Changes in HBV pgRNA levels after the initiation of cART. HBV pgRNA levels decreased after treatment in coinfected patients

Among the 32 subjects with detectable HBV pgRNA at baseline, HBV pgRNA was observed in 15 patients after 48 weeks of cART and became undetectable in eight individuals, six of whom were HBeAg‐negative. The median HBV pgRNA level in the remaining seven patients was 6.18 (IQR: 5.74–6.75) log_10_ copies/mL. Then, we evaluated the dynamics of HBV pgRNA levels during long‐term treatment. Twenty‐eight patients (28/32) were followed for a median interval of 138 (IQR: 54–240) weeks. At the end of the follow‐up time, HBV pgRNA became undetectable in 10 individuals, 7 of whom were HBeAg‐negative. And, HBV DNA suppression was achieved in these 10 subjects. In the remaining 18 subjects, the median HBV pgRNA level was 3.94 (IQR: 3.57–6.50) log_10_ copies/mL (Figure 6).

Among the 61 individuals who had undetectable levels of HBV pgRNA at baseline, 44 subjects who had serum samples had HBV pgRNA levels that were still below the LLD after a median of 126 (IQR: 48–312) weeks of follow‐up.

### 3.5 HBsAg loss and HBeAg loss

The enrolled patients were observed for 695.5 person‐years, and no HBsAg loss was observed. Thirteen achieved HBeAg clearance. No significant difference was observed in the median HBV pgRNA levels at baseline in patients who achieved HBeAg loss compared with those who did not (5.18 (IQR: 4.29–6.98) vs. 5.92 (IQR: 4.49–6.42) log_10_ copies/mL, *p* = 0.946). Notably, the proportion of patients with undetectable HBV pgRNA levels at baseline was higher in patients who achieved HBeAg clearance (33.33% vs. 0%, *p* = 0.033). HBV pgRNA became undetectable in 6 patients after a median 48 (IQR: 48–264) weeks of follow‐up in the HBeAg loss patients. However, Cox regression multivariate models showed that baseline HBsAg levels were associated with HBeAg loss (HR = 0.292 [95% CI: 0.115–0.740], *p* = 0.009), while baseline HBV pgRNA levels were not (Table 4).

**TABLE 4:** Factors associated with HBeAg clearance following cART

	Univariable	*p*	Multivariable	*p*
	OR (95% CI)		OR (95% CI)	
Gender				
Male	Reference			
Female	1.335 (0.349–5.104)	0.673		
Age (year)	1.026 (0.968–1.088)	0.392		
HBsAg (log_10_ IU/mL)	0.247 (0.096–0.633)	0.004	0.292 (0.115–0.740)	0.009
HBV DNA (log_10_ IU/mL)	0.695 (0.490–0.985)	0.041		
HBV RNA (log_10_ copies/mL)	0.799 (0.638–1.002)	0.052		
cART				
3TC‐based	Reference			
3TC + TDF‐based	10.786 (1.375–84.626)	0.024		
CD4^+^ T cell count				
<200 cells/μL	Reference			
>200 cells/μL	0.97(0.314–2.991)	0.957		
HIV RNA				
<5 log_10_ copies/mL	Reference			
>5 log_10_ copies/mL	1.003 (0.297–3.384)	0.996		

The transaminase and glycemic level in HIV/HBV coinfected patients were comparable during follow‐up period (Table 5). A total of 52, 50 and 23 patients underwent abdominal ultrasonography at baseline, after 96 weeks of cART, and after 240–480 weeks of cART, respectively. The abdominal ultrasonography report did not indicate any manifestations of HCC.

**TABLE 5:** Changes of biochemical markers pre‐ and post‐cART

	Baseline	*N*	2–3 years	*N*	5–6 years	*N*	7–10 years	*N*
ALT	29 (21–42.8)	132	31 (23–41)	128	26.9 (19–37)	85	27.4 (20.8–41)	48
AST	27.6 (23–36.6)	93	27 (23–33)	92	26 (22–30)	63	26.5 (23.5–32.2)	34
TBil	11.1 (8.4–15.1)	131	9.2 (6.4–11.9)	128	7.6 (5.7–10.4)	85	7.1 (5.6–8.8)	48
r‐GGT	23 (14–43.1)	120	44.8 (32.8–68.3)	126	43.5 (30–67)	84	43 (33.3–63.3)	44
ALP	82.4 (61.1–101.5)	89	103.5 (78.3–136)	92	101 (81.5–117)	63	98 (75–120)	31
Glu	5.1 (4.7–5.6)	120	5.1 (4.6–5.5)	128	5.1 (4.9–5.6)	84	5.2 (4.8–5.4)	48

### 3.6 HIV RNA suppression and CD4 ^+^ cell count improvements

**FIGURE 7:**
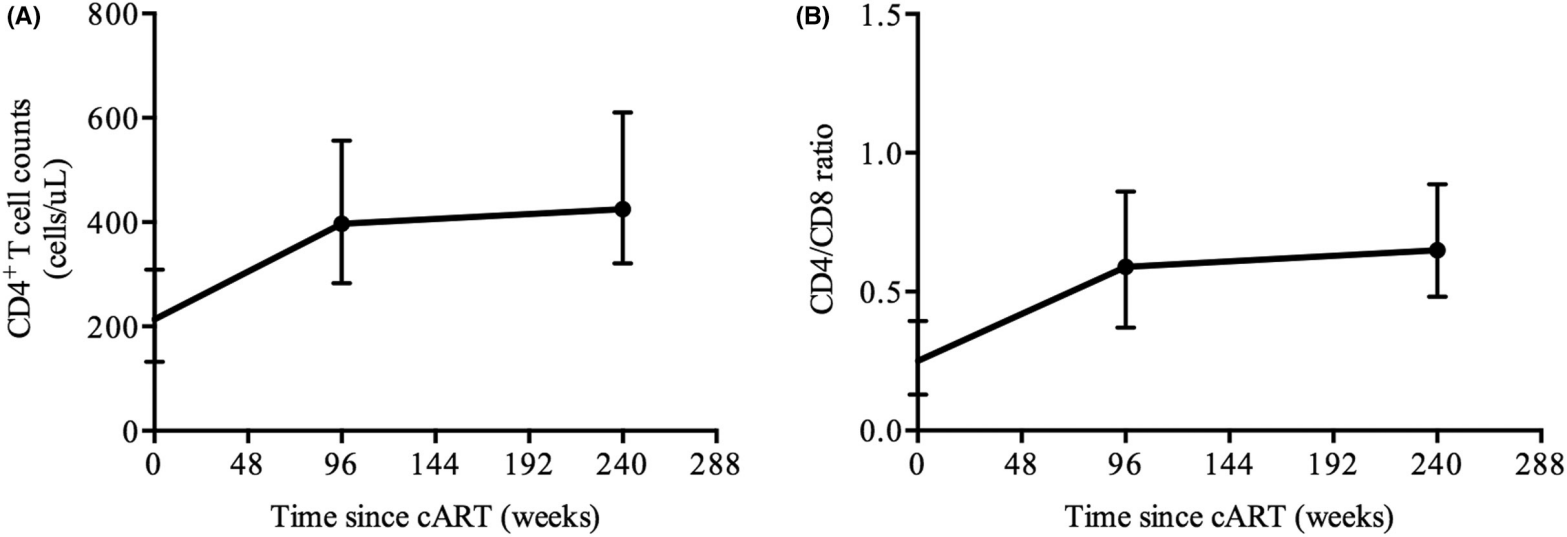
Recovery of T‐cell counts after the initiation of cART. The dynamics of CD4^+^ cell counts (A) and the CD4/CD8 ratio (B) after treatment in coinfected patients

We also evaluated the HIV RNA suppression and recovery of CD4 cell counts in HIV/HBV coinfected individuals after cART. HIV RNA < 20 copies/mL was achieved in 85.50% and 96.15% of patients at 96 and 240–480 weeks of treatment, respectively. The median CD4 cell counts were 397 (IQR: 283–556) cells/μL and 425 (IQR: 321–611) cells/μL, respectively (Figure 7).

## 4 DISCUSSION

This is the largest multicentre cohort study to evaluate the levels of HBV pgRNA changes in HIV/HBV coinfected subjects pre‐ and post‐cART in China. We demonstrated that 34.4% of cART‐naive HIV/HBV coinfected patients were positive for HBV pgRNA. HBeAg status and HBV DNA levels are two significant factors associated with undetectable HBV pgRNA levels. Additionally, the decrease in the HBV pgRNA level was more attenuated than the decrease in the HBV DNA level following cART, and HBV pgRNA is frequently undetectable in HBeAg‐negative patients after long‐term treatment. Notably, patients with undetectable HBV pgRNA levels are more likely to achieve HBeAg seroconversion.

HBeAg status is a marker of infectivity and of transcriptional activity of HBV.^19^ Therefore, HBV DNA and HBV pgRNA levels in HBeAg‐negative individuals are different from those in HBeAg‐positive subjects. Other studies reported that the HBV pgRNA levels in HBeAg‐negative patients were lower than those in HBeAg‐positive patients.^8^, ^20^, ^21^ In this study, the proportion of patients with undetectable levels of HBV pgRNA was significantly higher and the HBV pgRNA levels were lower in HBeAg‐negative patients than in HBeAg‐positive patients. Our multivariable analyses also demonstrate that HBeAg status is an independent factor associated with the proportion of patients with undetectable levels of HBV pgRNA. These results are in line with the results of a previous study.^20^ A possible explanation for these findings may be the lower transcriptional activity of cccDNA in HBeAg‐negative patients than that in HBeAg‐positive patients. This explanation is supported by the findings of the study conducted by Goncalves and his colleagues.^22^ Their study used a multiscale viral dynamic model to show that HBeAg‐negative patients had a lower rate of production of encapsidated pgRNA and a smaller basic reproduction number. Based on these results, we recommend dual HBV therapy for HBeAg‐positive coinfected patients when they initiate cART to effectively suppress HBV DNA and decrease HBV pgRNA levels.

It is worth mentioning that the proportion of patients with undetectable HBV pgRNA levels in our study was higher than that in previous study.^23^ This difference may be due to the normal or slightly elevated transaminase levels in the enrolled patients, in addition to the fact that most of the patients in this study were HBeAg‐negative. A study reported that ALT levels were positively associated with HBV pgRNA levels^24^ and other studies included mostly patients with elevated ALT levels,^23^ which may lead to higher HBV pgRNA levels than our results.

A relatively small sample size study demonstrated that HBV pgRNA levels were 4.57 (2.50–7.05) log_10_ IU/mL and were strongly correlated with HBV DNA in treatment‐naive HIV/HBV coinfected patients.^15^ The data from our study show that the HBV pgRNA level was 4.87 (4.19–6.11) log_10_ copies/mL in treatment‐naive HIV/HBV coinfected patients, which is similar to a previous report. We also show that HBV pgRNA levels are modestly correlated with HBV DNA and moderately correlated with HBsAg levels before treatment. However, these associations were not observed when patients were stratified by HBeAg status, which is consistent with the results of previous studies.^10^, ^15^ Notably, a few studies found that the positive correlation between HBV pgRNA and HBsAg remained in HBeAg‐positive patients but was absent in HBeAg‐negative patients,^9^, ^24^ indicating that other factors, such as the host immune response, may also play an important role in the maintaining the correlation.

During long‐term NA treatment, HBV DNA replication is suppressed. Our study found that 88.4% of patients achieved HBV DNA < 20 IU/mL after 240 weeks of cART, which supports the findings of a previous study on HIV/HBV coinfection.^5^ However, it is quite difficult to completely eradicate chronic HBV infection due to the long‐term presence of intrahepatic HBV cccDNA. As a marker reflecting the activity of cccDNA, studies have demonstrated that HBV pgRNA levels were associated with the failure to achieve HBeAg clearance and that HBV DNA levels rebounded after NA cessation in CHB patients.^12^, ^23^ Therefore, monitoring the dynamics of HBV pgRNA levels after cART is important for evaluating the antiviral efficacy of treatment in HIV/HBV coinfected patients. In this study, we demonstrated that 10 patients achieved undetectable HBV pgRNA levels, and most of them were HBeAg‐negative during the follow‐up period, indicating a significant decline in the active transcription of cccDNA. Most of them still had detectable HBV pgRNA, demonstrating that they should not stop anti‐HBV treatment even though they achieved HBV DNA suppression for a long time. Therefore, cabotegravir and rilpivirine may not be suitable for them. HBeAg loss occurred in 13 patients. They had lower baseline HBV pgRNA and approximately 50% of them had undetectable HBV pgRNA levels during follow‐up, indicating that HBV pgRNA levels may be correlated with HBeAg clearance in HIV/HBV coinfected patients. Unfortunately, the Cox regression multivariate model showed that HBV pgRNA at baseline was not associated with HBeAg loss, which may be due to the small number of patients with HBeAg loss. Future studies could enlarge the sample size to verify our conclusion.

As long‐acting drugs, cabotegravir and rilpivirine are noninferior to standard TDF combined with 3TC‐containing therapy for maintaining the HIV RNA suppression rate in patients without active HBV infection.^16^, ^17^ However, cabotegravir and rilpivirine regimens do not contain any active anti‐HBV agents. There were 18 patients who still had detectable HBV pgRNA levels during the long‐term follow‐up period in our study, which means HBV DNA may rebound after NAs cessation. Therefore, cabotegravir and rilpivirine may not be suitable for these patients when they want to discontinue oral therapy and switch to long‐acting therapy.

Some limitations exist in this study. First, the number of HBeAg‐positive HIV/HBV coinfected patients was small in this study, thus, future studies should pay more attention to HBeAg‐positive patients to verify the present study. Second, HBV genotype analysis was not conducted in this study, thus we were not able to evaluate the impact of the HBV genotype on HBV pgRNA levels. Finally, no liver biopsies were performed to measure intrahepatic cccDNA, so we could not explore the correlations between HBV RNA and intrahepatic cccDNA levels.

## 5 CONCLUSIONS

In conclusion, our study reveals the HBV pgRNA distribution in HIV/HBV coinfected Chinese patients from a multicentre cohort pre‐ and post‐cART. After cART initiation, HBV pgRNA is still detectable even if HBV DNA is suppressed in most HBeAg‐positive patients and undetectable HBV pgRNA levels may be associated with HBeAg seroconversion. Based on these results, we suggest that anti‐HBV treatment is still needed for HIV/HBV coinfected patients with detectable HBV pgRNA even after long‐term cART.

## AUTHOR CONTRIBUTIONS

LX performed laboratory experiments, acquired, analysed all the data and drafted the manuscript; XDL, LFL, XSL, YH and TZ performed laboratory experiments; XJS, YLL and WC collected the clinical data; TSL designed the study, evaluated and interpreted data, obtained funding; All authors participated in the manuscript review and approved the final version of the text.

## CONFLICT OF INTEREST

The authors declare that they have no competing interests.

## ETHICAL APPROVAL

The Ethics Committee of Peking Union Medical College Hospital approved this study and all the participants provided written informed consent.

## Data Availability

The data that support the findings of this study are available from the corresponding author upon reasonable request.
